# A Na_2_CO_3_-Responsive Chitinase Gene From *Leymus chinensis* Improve Pathogen Resistance and Saline-Alkali Stress Tolerance in Transgenic Tobacco and Maize

**DOI:** 10.3389/fpls.2020.00504

**Published:** 2020-04-28

**Authors:** Xiangguo Liu, Ying Yu, Qing Liu, Suren Deng, Xuebo Jin, Yuejia Yin, Jia Guo, Nan Li, Yang Liu, Siping Han, Chuang Wang, Dongyun Hao

**Affiliations:** ^1^Instutute of Agricultural Biotechnology, Jilin Academy of Agricultural Sciences, Changchun, China; ^2^School of Basic Medicine, Guizhou University of Traditional Chinese Medicine, Guiyang, China; ^3^Key Laboratory of Arable Land Conservation (Middle and Lower Reaches of Yangtze River), Ministry of Agriculture (MOA), Huazhong Agricultural University, Wuhan, China; ^4^Microelement Research Center, College of Resources & Environment, Huazhong Agricultural University, Wuhan, China

**Keywords:** chitinase, saline-alkali stress, biotic stress, *Leymus chinensis*, tobacco, maize

## Abstract

Salinity and microbial pathogens are the major limiting factors for crop production. Although the manipulation of many genes could improve plant performance under either of these stresses, few genes have reported that could improve both pathogen resistance and saline-alkali stress tolerance. In this study, we identified a new chitinase gene *CHITINASE 2* (*LcCHI2*) that encodes a class II chitinase from *Leymus chinensis*, which grows naturally on alkaline-sodic soil. Overexpression of *LcCHI2* increased chitinase activity in transgenic plants. The transgenic tobacco and maize exhibited improved pathogen resistance and enhanced both neutral salt and alkaline salt stress tolerance. Overexpression of *LcCHI2* reduced sodium (Na^+^) accumulation, malondialdehyde content and relative electrical conductivity in transgenic tobacco under salt stress. In addition, the transgenic tobacco showed diminished lesion against bacterial and fungal pathogen challenge, suggesting an improved disease resistance. Similar improved performance was also observed in *LcCHI2*-overexpressed maize under both pathogen and salt stresses. It is worth noting that this genetic manipulation does not impair the growth and yield of transgenic tobacco and maize under normal cultivation condition. Apparently, application of *LcCHI2* provides a new train of thought for genetically engineering saline-alkali and pathogen resistant crops of both dicots and monocots.

## Introduction

The biotic and abiotic stresses, such as pathogens and salinity, severely affected crop growth and agricultural productivity worldwide. According to incomplete statistics of UNESCO and FAO, 950 million ha (6.4%) of the world’s land area has saline-alkali soil, and about 10% of this area is found in China. The alkaline soils of China contains high levels of Na_2_CO_3_ and NaHCO_3_ (Wang et al., 1993). Only few species of plants could grow in such soils and they are sparsely distributed. *Leymus chinensis*, a perennial grass of family gramineae, grows naturally on alkaline soil, suggesting the existence of such a mechanism. Apparently, identification of functional gene(s) specifically responsible for stress tolerance would be a prerequisite of crop improvement through biotech breeding.

Chitinase is one of the important enzyme family that has been found to involve in such a mechanism of divergent biological functions. Chitinases (EC 3.2.1.14) are a family of glycosyl hydrolases (GHs) responsible for the hydrolysis of the chitin polymer (a β-1,4-linked *N*-acetylglucosamine), a structural component found in the cell walls of fungi, insects, a variety of crustaceans and nematode eggs (Kesari et al., 2015). Interestingly, although the chitin is not present in plants, different subgroups of chitinase genes were identified in plants (Kesari et al., 2015). Plant chitinase proteins are generally divided into six classes, I to VI (Patil et al., 2000). The classes III and V belong to the GH18 family, whereas class I, II, IV, and VI belong to the GH19 family (Patil et al., 2000). Both families exhibit diversity in their nucleic acid sequences, protein structures, substrate specificities, sensitivity to inhibitors and mechanisms of catalysis. Along with active chitinases, the plant genome also consists of a large number of sequences encoding catalytically inactive chitinases that are referred to as chitinase-like (CTL) proteins (Kesari et al., 2015). A few studies suggested that the likely substrates of plant CTL proteins may be arabinogalactan protein, chitooligosaccharides, *N*-acetylchitooligosaccharides and other GlcNAc-containing glycoproteins or glycolipids, but the substrates of most plant CTL proteins remain uncertain (van Hengel et al., 2001; Zhong et al., 2002; Sanchez-Rodriguez et al., 2012). The intrinsic diversities of plant chitinase imply that the chitinase/CTL genes likely have a broad function.

Because chitin is the major component of fungal cell walls, earlier studies on the role of chitinase genes focused extensively on its involvement in plant defense responses to fungal pathogen infection (Brogue et al., 1991; Verburg and Huynh, 1991). Chitinase gene expression in plant tissues is strongly induced by fungal pathogens and chitin oligosaccharides (Hong and Hwang, 2002; Seo et al., 2008). Interestingly, the expression of chitinase gene responded also to infection of viruses, bacteria and oomycetes, which do not have chitin or related structures in their cell wall (Hong and Hwang, 2006; van Loon et al., 2006). Accordingly, a number of plant chitinases are defined as classic pathogenesis-related proteins (PRs) including PR-3, -4, -8, and -11 families (van Loon et al., 2006). Overexpression of these PR proteins conferred resistance to plant disease in different plant species (Brogue et al., 1991; Nishizawa et al., 1999; Tabaeizadeh et al., 1999; Yamamoto et al., 2000; Takahashi et al., 2005; Shin et al., 2008).

Plants chitinase and CTL genes play a role not only in defense related processes but also in abiotic stress tolerance. In *Arabidopsis thaliana*, the *AtPR3* gene which encodes a class II chitinase was induced by high salt (Seo et al., 2008). The pepper class II basic chitinase gene *CaChi2* was induced by salt, drought and osmotic stresses (Hong and Hwang, 2002, 2006). The expression of *ptch28* from *Lycopersicon chilense* plants induced by osmotic and abscisic acid (Chen et al., 1994). In addition to salt, osmotic and drought stresses, expression of the class II chitinase gene from Bermuda grass was reported to be up-regulated by cold stress and function as an antifreeze protein (Nakamura et al., 2008). Similarly, in winter rye (*Secale cereal*) and highbush blueberry, cold stress induced the chitinase like proteins, which showed antifreeze activity *in vitro* (Yeh et al., 2000; Kikuchi and Masuda, 2009). Interestingly, most of the reported chitinase genes that responsive to both biotic and abiotic stresses belonged to the class II family and homolog to PR3 proteins (Supplementary Figure S1). Furthermore, overexpression of these PR3 genes, such as *AtPR3* and *CaChi2*, significantly increased the salt resistance of the transgenic plants (Hong and Hwang, 2006; Seo et al., 2008). In contrast, mutation of the *AtCTL1* resulted in oversensitive to salt and drought stresses (Kwon et al., 2007).

Wildly grown plants have developed through evolution into adaptive mechanisms against the various environmental threatening. Apparently, identification of functional gene(s) specifically responsible for stress tolerance would be a prerequisite of crop improvement through biotech breeding. In a previous report, a chitinase like EST sequence was isolated from *Leymus chinensis* which was treated with Na_2_CO_3_ (Jin et al., 2006). Accordingly, we cloned a unique chitinase gene and designated it as *LcCHI2*, which belongs to class II chitinase gene family. In *Leymus chinensis*, expression of *LcCHI2* was induced by salt stress leading to an increase in chitinase activity. Overexpression of *LcCHI2* increased pathogen and salt stress resistance in both tobacco and maize. This study provides a novel train of thought in improvement of both pathogen resistance and salt stress tolerance, in particular alkaline salt stress, through overexpression of a single gene in dicots and monocots.

## Materials and Methods

### Cloning and Expression Analysis of *LcCHI2*

Total RNA was extracted from 4-week-old seedlings of *Leymus chinensis* treated with 100 mM Na_2_CO_3_ using RNAiso plus (TaKaRa, Japan) according to the instruction manual. Full-length cDNA of LcCHI2 sequence was obtained using a SMART^TM^ RACE cDNA Amplification Kit (Clontech, United States) for 3′ RACE with the primers 5′-CCGACCAGTTCCAATGGGGCT-3′ and 5′-GGCCACGCGTCGACTAGTACTTTT TTTTTTTTT TTTT-3′.

For expression analysis of *LcCHI2*, total RNA was extracted from 4-week-old *Leymus chinensis* harvested at different time points under various stress treatments. A 771 bp fragment of *LcCHI2* cDNA was amplified in 30 cycles with the primers 5′-ATGGCGAGGTTTGCTGCCCTCG-3′ and 5′-CTAGCTAGCGAAGTTTCGCTGGGT G-3′. As an internal standard for cDNA levels, a 245 bp fragment of ACTIN cDNA (GenBank accession number AB181991) was amplified simultaneously in 28 cycles with primers 5′-GGACCTTGCTGGTCGTGACC-3′ and 5′-CCTCAGGGCACCTGAACCTT T-3′. DIG High Prime DNA Labeling and Detection Starter Kit I (Roche, United States) was used for Northern blot analysis. The specific *LcCHI2* cDNA fragment was amplified with the primers 5′-ATGGCGAGGTTTGCTGCCC-3′ and 5′-CTAGCTAGCGAAGTTTCGCTGGG-3′ and then labeled with DIG-High Prime used for the hybridization probe.

## Agrobacterium-Mediated Tobacco and Maize Transformation

The *LcCHI2* gene was amplified with the primers 5′-ccggaattcATGGCGAGGTTTGCTGCCCTCG-3′ and 5′-cgcggatccCTAGCTAGCGAAGTTTCGCTGGGTG-3′. The PCR products were digested with *Eco*R I/*Bam*H I and cloned into the *pUC119-pCaMV35S-tNOS* vector to generate *pUC119-pCaMV35S::LcCHI2-tNOS*. The fragment of *pCaMV35S::LcCHI2-tNOS* was collected by *Hin*d III digestion and cloned into pBI121vector. The final construct map was shown in Supplementary Figure S1A. After sequencing the positive clones, the final construct pBI121-*pCaMV35S*::*LcCHI2*-tNOS were transformed into *Agrobacterium* strain EHA105. Transgenic tobacco (*Nicotiana tabacum*) plants were produced by using *Agrobacterium*-mediated transformation as described by the previously reported method (Hoekema et al., 1983). Transgenic plants were selected on MS medium containing 50 mg/ml kanamycin and 500 mg/ml carbenicillin.

For maize transformation, the sequence of *LcCHI2* from *pBI121-pCaMV35S::LcCHI2-tNOS was* digested with *Spe*I/*Bam*H I and cloned into the *pTF101-pZmUBI-tNOS* vector to generate *pTF101-ZmUBI::LcCHI2-tNOS*. The final construct map was shown in Supplementary Figure S1B. Immature zygotic embryos of HiII (B73 × A188) were infected with *Agrobacterium* strain EHA105 harboring the binary vector *pTF101-ZmUBI::LcCHI2-tNOS*. Transgenic plants were selected and produced by the previously reported method (Frame et al., 2002).

### Chitinase Activity Assay

Chitinase activity was measured in 10 mM potassium phosphate buffer (pH 7.0) containing 0.1 mM 4-methylumbelliferyl-*β*-D-*N*, *N*′-diacetylchitobiose (Sigma, Germany) as a substrate by the previously reported method (O’Brien and Colwell, 1987). One unit of chitinase activity was defined as the amount of enzyme required to produce 1 μmol of 4-methylumbelliferone per minute.

### Measurement of Malondialdehyde (MDA) Contents and Ion Leakage Ratio

Fresh leaves (0.3 g) were ground properly in 5 ml of 5 % trichloroacetic acid solution and centrifuged for 10 min at 3000 rpm. 2 ml of the supernatant was reacted with 2 ml 0.67% thiobarbituric acid and then it was heated for 30 min, at 100°C in a water bath and then immediately cooled on ice. After centrifugation for 10 min, at 3000 rpm, the absorbance of the supernatant was read at 450, 532, and 600 nm. The contents of MDA were calculated using the formula: MDA concentration (μmol/L) = 6.45 × (A_532_−A_600_) − 0.56 × A_450_).

Ion leakage ratio was measured as relative electrical conductivity parameter. 0.1 g leaves were sampled from different plants, rinsed briefly with deionized water and immediately placed into a tube with 10 mL of deionized water. Conductivity (I1) was measured using an electroconductivity meter (model 1054, VWR Scientific, Phoenix) after the tubes were placed at 22°C overnight. Then, the samples were heated at 100°C for 30 min and conductivity (I2) was measured again. Relative electrical conductivity was expressed as (I1/I2) × 100%.

### Sodium Content

Seedings of wild type (WT) and T2 generation transgenic tobacco plants were cultured in nutrient solution for 1 month and then treated with 0 or 200 mM NaCl. After 6 days of treatment, the shoots and roots were oven dried, digested with H_2_SO_4_-H_2_O_2_. The digest was dissolved in deionized water and sodium content was estimated by flame spectrophotometer (M410, Waters Corporation, America).

### Salt Stress Treatments

The plant seedlings of *Leymus chinensis* were grown in vermiculite with a light/dark cycle of 16 h/8 h at 25°C. They were irrigated with one-half-diluted Murashige and Skoog (MS) medium every 3 days. Four-week-old seedlings of *Leymus chinensis* were treated with 400 mM NaCl and 100 mM Na_2_CO_3_, respectively. Leaves of treated plants were harvested up to 0, 6, 12, and 24 h, respectively, by rapid freezing in liquid nitrogen and kept at −80°C for further analysis.

To determine abiotic stresses tolerance of the transgenic tobacco on disc, seeds of wild-type and T2 generation transgenic tobacco were sowed on MS medium containing 200 mM NaCl, 30 mM Na_2_CO_3_ or 500 mM sorbitol, respectively. For soil experiment, tobacco seeds germinated in soil cultured with a light/dark cycle of 16 h/8 h at 25°C. Thirty plants for each line were used for salt and drought stress treatment, respectively. For salt stress, nearly 3-week-old plants were irrigated for 25 days with either tap water or tap water containing 400 mM NaCl or 100 mM Na_2_CO_3_.

To determine abiotic stresses tolerance of the transgenic maize, the transgenic events with HiII background (CHI-Ox2, CHI-Ox5, and CHI-Ox7) were backcross to local commercial inbred line X923-1. The segregated positive and negative BC5F2 plants were grown in pots (10 × 10 cm) containing peat: vermiculite (5:1, v/v) medium in a greenhouse with a light/dark cycle of 16 h/8 h at 30°C. PCR analysis was used to screen for positive transformants, and 2-week-old seedlings were water with either tap water or tap water containing 200 mM NaCl or 50 mM Na_2_CO_3_ for 6 days. Photograph was recorded after each treatment by a camera.

### Pathogen Response Assays of Transgenic Tobacco and Maize

For bacterial infection analysis, *Pseudomonas tabaci (Wolf et Foster) Stevens* was grown in NBY medium and bacterial cells were collected, washed and resuspended in 10 mM MgSO_4_. The density of bacterial cells was determined by measuring absorbance at OD_600_. Bacterial cells in suspension were infiltrated into fully expanded 6-week-old tobacco leaves using a 1 ml plastic syringe without a needle. After 5 days, the average lesion area for each independent transgenic line was calculated and compared with that of wild-type tobacco palnts.

For fungal resistance analysis, *Alternaria alternata (Fries) Keissler* was cultured on potato dextrose agar medium at 28°C. When the mycelia reached the edge of the plate, 0.5 cm diameter agar discs were excised from the edge of growing colonies using a cork borer and inverted onto the detached leaves from wild-type and transgenic tobacco plants. All leaves were placed on wet filter paper in Petri dishes and incubated at 28°C to permit normal disease development under high humidity. After 7 days, the average lesion area for each independent transgenic line was calculated and compared with that of wild-type tobacco plants.

Maize pathogen *Exserohilum turcicum* and *curvularia lunata* (Wakker) Boed were provided by Iinstitute of plant protection, Jilin academy of agricultural sciences. *Exserohilum turcicum* and *curvularia lunata* (Wakker) Boed were cultured on potato dextrose agar medium at 28°C. When the mycelia reached the edge of the plate, 0.6 cm diameter agar discs were excised from the edge of growing colonies using a cork borer and inverted onto the detached leaves from transgenic and null control maize plants. All leaves were placed on wet filter paper in Petri dishes and incubated at 28°C to permit normal disease development under high humidity. After 3 days, the average lesion area for each independent transgenic line was calculated.

### Nucleotide and Protein Sequences Accession Numbers

The nucleotide and protein sequences reported in this paper have been deposited in the Genbank nucleotide database and protein database under the accession numbers GQ397277 and ACV20870.

## Results

### Cloning and Characterization of *LcCHI2* From *Leymus chinensis*

According to the EST sequence information from literature (Jin et al., 2006), we cloned a full-length cDNA of the target chitinase gene by 3‘RACE technology from *Leymus chinensis*, and was designated as *LcCHI2* (GenBank accession number GQ397277). Sequence analysis revealed that *LcCHI2* contains a 771 bp open reading frame encoding a polypeptide of 256 amino acids. Pfam scan showed that the deduced protein is a class II chitinase belonging to the GH19 family (PF00182)^1^. To obtain more information of the LcCHI2 protein, a phylogenetic tree was constructed including LcCHI2 orthologes and all the class II chitinases of *Arabidopsis*. LcCHI2 belonged to a cluster including three pathogenesis-related protein 3 (HvPR3, TaPR3, and AtPR3) and two chitinases (ScCHI46, CaCHI2) (Supplementary Figure S2). LcCHI2 showed more than 95% homology to HvPR3, TaPR3 and ScCHI46 and 71% homology to CaCHI2 (Supplementary Figure S3A). Multiple protein alignment revealed two conserved chitinase family 19 signature domains in all the five orthologes (Supplementary Figure S3B). However, an *N*-terminal secretive signal peptide only found in LcCHI2 and its monocot orthologes (Supplementary Figure S3B).

### Sodium Stress Induced the Expression of *LcCHI2* and Chitinase Activity in *Leymus chinensis*

**FIGURE 1:**
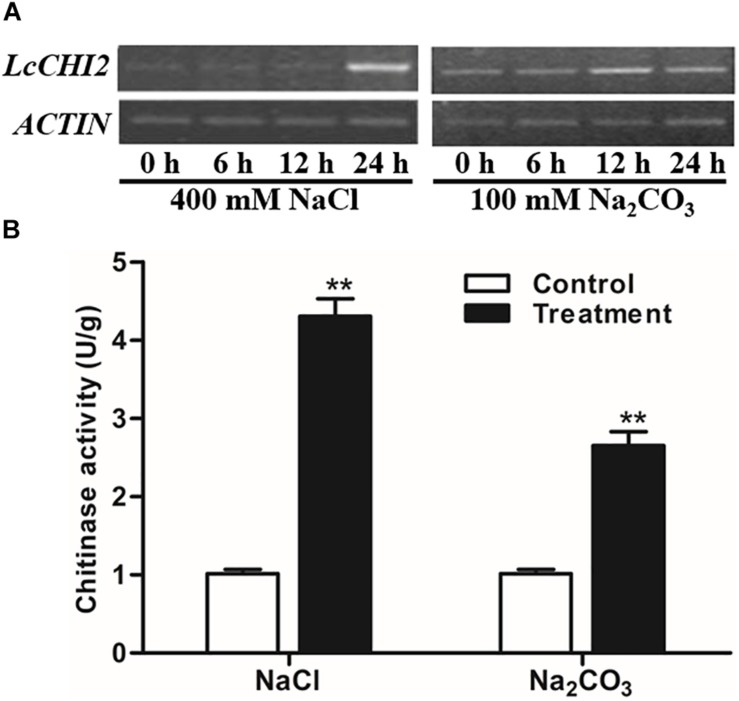
Regulation of *LcCHI2* and chitinase activity under salt stress conditions in *Leymus chinensis*. **(A)** Expression analysis of the *LcCHI2* after treatment with 400 mM NaCl and 100 mM Na_2_CO_3_ for the indicated times. Total RNA were extracted from 4-week-old seedlings of *Leymus chinensis* following treatments as indicated and reverse transcribed. The cDNA were used as templates for RT-PCR and the *ACTIN* gene was amplified as an internal control. The PCR products were examined by electrophoresis in 1% (w/v) agarose gel. **(B)** Chitinase activity assay under salt stress conditions in *Leymus chinensis*. Enzyme activity assays were carried out with the leaves of *Leymus chinensis* after treatment with 400 mM NaCl and 100 mM Na_2_CO_3_ for 24 h. Values are the mean ± SE obtained from three biological replicates. ** are significantly different at *P* < 0.01 (LSD test).

As the EST of *LcCHI2* was isolated from alkaline-sodic stressed tissues, we measured the expression of *LcCHI2* in *Leymus chinensis* under different abiotic conditions. A significantly induction of *LcCHI2* transcripts was observed under 400 mM NaCl after 24 h treatment comparing with the control plant (Figure 1A). Similarly, the expression of *LcCHI2* increased more than 2-fold under 100 mM Na_2_CO_3_ after 12 and 24 h treatment compared with the control plant (Figure 1A). Subsequently, the chitinase activities were determined under NaCl and Na_2_CO_3_ conditions after 24 h treatment. The results showed that chitinase activities increased by 4.3- and 2.6-fold after 24 h exposure to 400 mM NaCl and 100 mM Na_2_CO_3_, respectively (Figure 1B). In contrast, the expression of *LcCHI2* and chitinase activity were not changed in *Leymus chinensis* under 20% PEG condition in 24 h (data not show).

### Overexpression of *LcCHI2* in Tobacco Enhanced Plant Tolerance to Salt Stress

**FIGURE 2:**
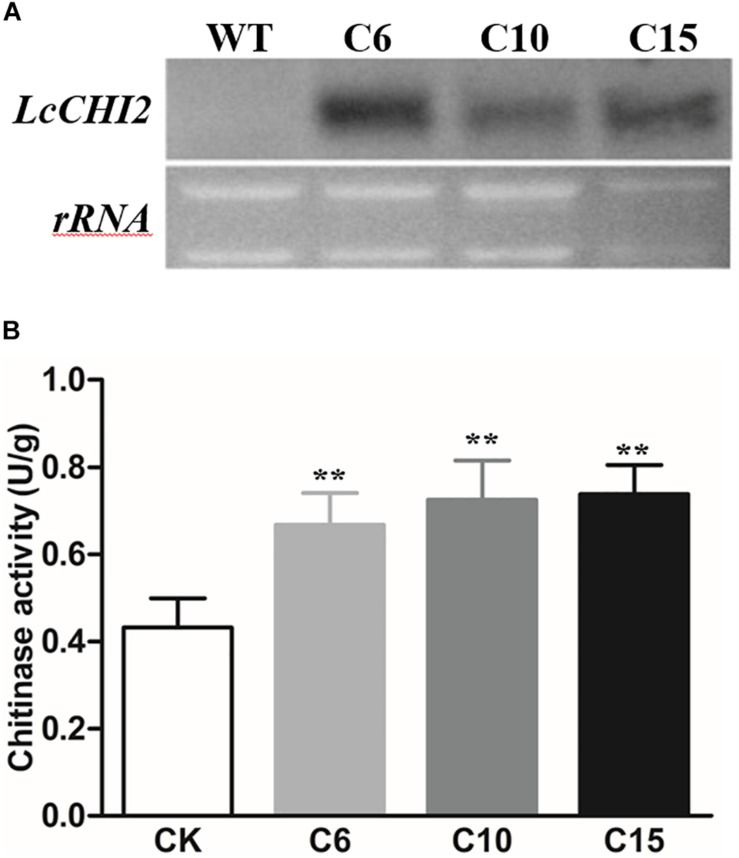
**(A)** Northern blot analysis of *LcCHI2* transcripts in wild-type (WT) and transgenic tobacco plants (C6, C10, C15). A DIG-labeled LcCHI2 probe was used for hybridization. **(B)** Chitinase activity of WT and transgenic tobacco plants. Values are the mean ± SE obtained from three biological replicates. ** are significantly different at *P* < 0.01 (LSD test).

To further investigate the function of *LcCHI2*, transgenic tobacco plants that constitutively express the *LcCHI2* gene under the control of the CaMV 35S promoter were developed by agrobacterium-mediated transformation. Twenty one independent positive transgenic lines were selected by kanamycin resistance. Three represented lines that showed high expression of *LcCHI2* were selected for further analysis (Figure 2A). Moreover, chitinase activity increased by 1.5–1.7-fold over WT plants in the selected transgenic lines (Figure 2B). Comparing with the WT plants, the *LcCHI2*-overexpression plants exhibited no obvious phenotypic difference from the WTs under normal growth conditions (Supplementary Figure S4).

**FIGURE 3:**
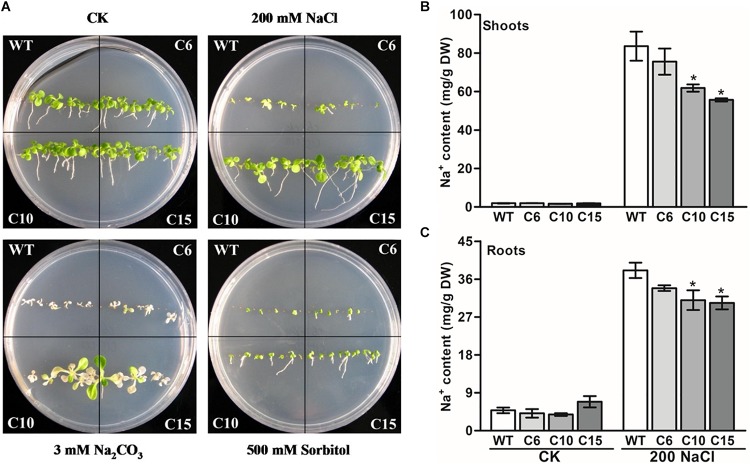
Abiotic stress tolerance assays of wild-type and *LcCHI2*-overexpressed plants in tobacco. **(A)** The WT and *LcCHI2* overexpression (C6, C10 and C15) plants were grown on MS agar plates containing 200 mM NaCl, 30 mM Na_2_CO_3_, and 500 mM sorbitol for 30 days. **(B, C)** Na^+^ content of wild-type and *LcCHI2* overexpression plants in shoots and roots. Values are the mean ± SE obtained from three biological replicates. * are significantly different at *P* < 0.05 (LSD test).

As the expression of *LcCHI2* is induced by salt stress, phenotypes of the WT and *LcCHI2*-overexpression lines were analyzed on MS medium containing 200 mM NaCl, 30 mM Na_2_CO_3_ and 500 mM sorbitol, respectively. No difference was observed between WT and *LcCHI2*-overexpression plants growth on normal MS medium. However, the *LcCHI2*-overexpression plants survived well under salt stress (NaCl and Na_2_CO_3_) in comparison with WT plants, especially the C10 and C15 lines (Figure 3A). In contrast, the *LcCHI2*-overexpression plants only exhibited a minor effect for osmotic stress (Figure 3A). To further evaluate the abiotic resistance of *LcCHI2*-overexpression plants, soil growth plants were grown under salt and drought stress conditions. There were no observed differences in phenotypes between *LcCHI2*-overexpression and WT plants, when they were grown under normal and drought stress conditions (Supplementary Figure S5A). However, 6 days after watering 400 mM NaCl or 100 mM Na_2_CO_3_, the wild-type seedlings showed wilting phenotypes and ultimately died, whereas the transgenic tobacco plants continued to grow (Supplementary Figures S5B,C).

Because the overexpression of *LcCHI2* increased salt stress tolerance other than osmotic or drought stresses, sodium contents were determined in the roots and shoots of WT and transgenic plants under normal and NaCl treatment conditions. As expected, NaCl treatment significantly increased Na^+^ content in the shoots and roots of WT and transgenic plants (Figures 3B,C). The overexpression of *LcCHI2* decreased the Na^+^ content from 10 to 33% in the shoots and from 11 to 22% in the roots of transgenic lines compared with the corresponding WTs under NaCl treatment condition (Figures 3B,C). Meanwhile, the Na^+^ content showed a significantly decrease only in transgenic lines C10 and C15 other than line C6, which is coincident with the strong resistance to sodium stress of the transgenic lines C10 and C15 (Figure 3A).

Malondialdehyde (MDA) content and relative electrical conductivity are widely used as indicators for lipid peroxidation and the degree of plant cell injury under stress treatment, respectively. There are no obvious differences in MDA content and relative electrical conductivity between WT and *LcCHI2* overexpression plants under normal growth condition. Although 24 h salt treatment significantly increased the MDA content and relative electrical conductivity in both WT and *LcCHI2* overexpression plants, the transgenic lines showed a lower MDA content and relative electrical conductivity compared with WT under salt stress condition (Supplementary Figure S6).

### Overexpression of *LcCHI2* Conferred Pathogen Tolerance in Tobacco

**FIGURE 4:**
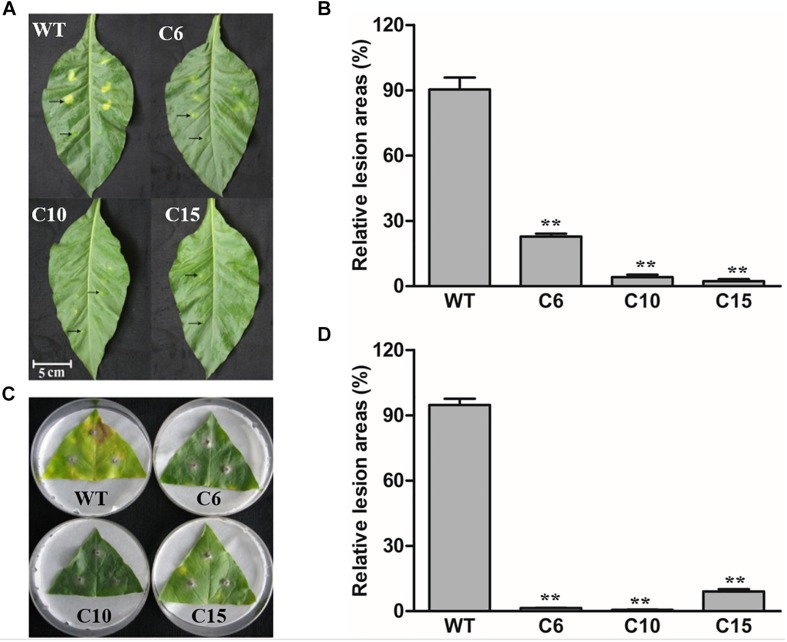
Pathogenesis analysis of *LcCHI2*-transgenic tobacco. **(A, B)** Resistance of transgenic tobacco to the bacterial pathogen *Pseudomonas tabaci* (*Wolf and Foster*) *Stevens*. Fully expanded leaves of tobacco plants were syringe-infiltrated with 0.1 ml solution of *P. tabaci*. Arrows in the upper line represent the sites inoculated with bacteria and arrows in the lower line represent the sites with mock-inoculated. The average lesion area of each independent transgenic line was calculated and their relative lesion areas are shown in columns after comparison with the average lesion area on wild-type tobacco. The photograph was taken 5 days after inoculation. **(C, D)** Responses of transgenic tobacco to the fungal pathogen *Alternaria alternata (Fries) Keissler*. Detached leaves were challenged with mycelia of *A. alternata*. The average lesion area of each independent transgenic line was calculated and their relative lesion areas are shown in columns after comparison with the average lesion area on wild-type tobacco. The photograph was taken 7 days after inoculation. Values are the mean ± SE obtained from three biological replicates. ** are significantly different at *P* < 0.01 (LSD test).

As a homolog of PR3 proteins, LcCHI2 is presumed to be involve in pathogen resistance too. Thus, the *LcCHI2*-overexpression plants were inoculated with bacterial and fungal pathogens, respectively. Leaves of WT and *LcCHI2*-overexpression tobacco plants were inject-inoculated with spore suspensions of the bacterial pathogen *Pseudomonas tabaci (Wolf et Foster) Stevens* and symptom development was subsequently monitored for 5 days. The examined disease symptoms included chlorosis and necrosis expansion surrounding the primary infection sites. As shown in Figure 4, WT tobacco plants were more sensitive to bacterial infection than *LcCHI2*-overexpression tobacco plants in detached leaves, in which pathogen infection resulted in significantly reduced disease symptoms. The leaf lesion size of WT tobacco plants was 5- to 10-fold larger than that of *LcCHI2*-overexpression tobacco plants (Figures 4A,B). Resistance to the fungal pathogen *Alternaria alternata (Fries) Keissler* was also identified in *LcCHI2*-overexpression tobacco plants by a detached leaf inoculation test after 7 days inoculation. WT tobacco plants showed typical necrosis symptoms surrounded by chlorotic halos and extensive pathogen sporulation, while transgenic lines had significantly smaller lesion sizes than wild-type tobacco (Figures 4C,D). The enhanced resistance to *Pseudomonas tabaci and Alternaria alternata* in transgenic tobacco demonstrated that disease resistance conferred by *LcCHI2* overexpression is effective against both bacterial and fungal pathogen.

### Overexpression of *LcCHI2* in Maize Enhanced Tolerance to Salt and Pathogen Stresses

**FIGURE 5:**
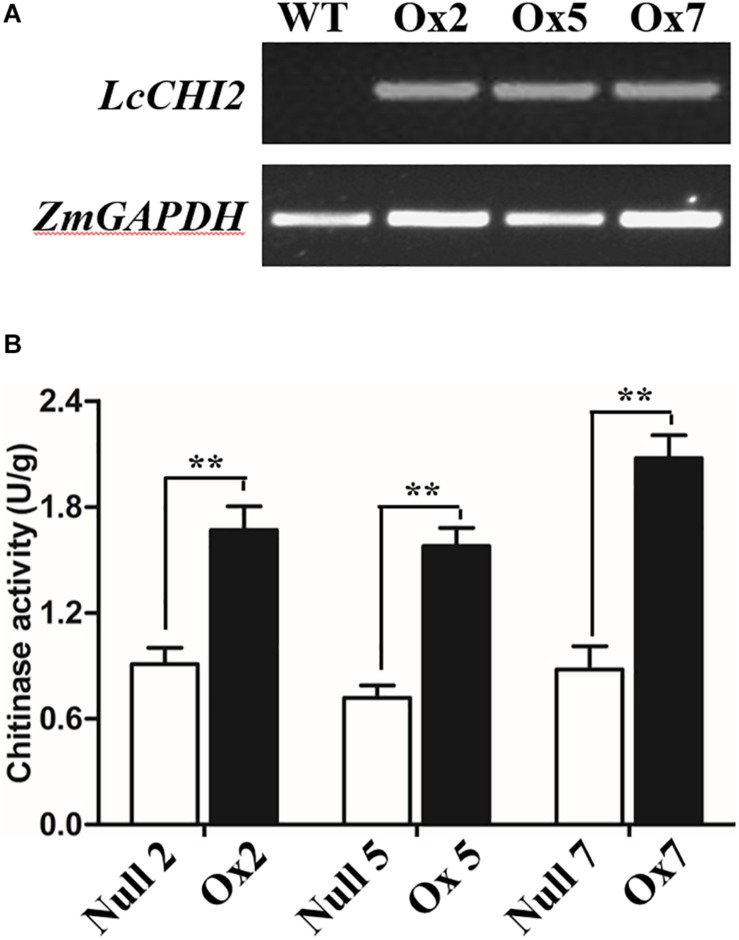
**(A)** RT-PCR analysis of *LcCHI2* transcript in wild-type (WT) and transgenic maize plants (Ox2, Ox5, Ox7). Total RNA were extracted from 2-week-old seedlings of positive and null transgenic plants. The cDNA were used as templates for RT-PCR and the actin gene was amplified as an internal control. The PCR products were examined by electrophoresis in 1% (w/v) agarose gel. **(B)** Chitinase activity of null and transgenic maize plants. Values are the mean ± SE obtained from three biological replicates. ** are significantly different at *P* < 0.01 (LSD test).

**FIGURE 6:**
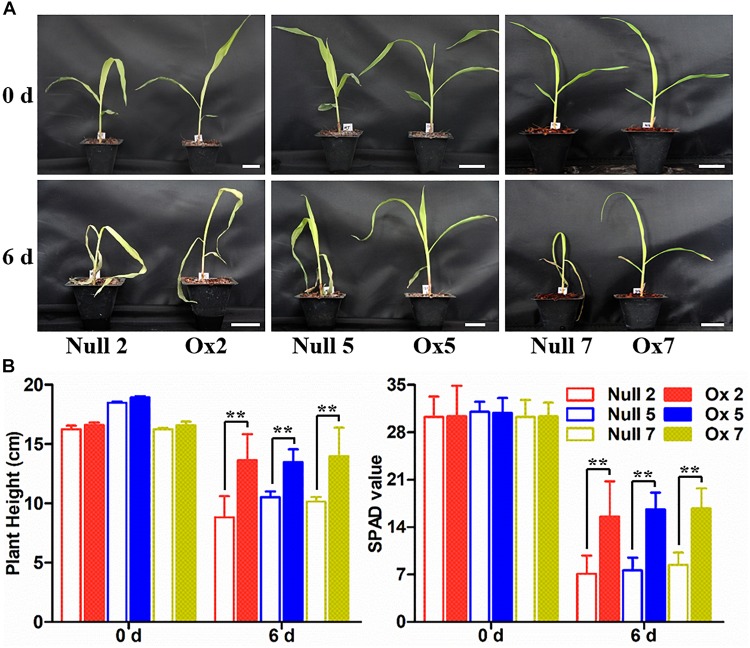
Salt stress tolerance assays of null and *LcCHI2* overexpression plants in maize. **(A)** Soil growth null and *LcCHI2* overexpression plants were watered with 200 mM NaCl for 6 days. **(B)** Plant height and SPAD value of null and *LcCHI2* transgenic maize were measured every day. The transgenic events with HiII background were backcross to local commercial inbred line X923-1. The segregated positive and negative BC5F2 plants were used for the treatment. Values are the mean ± SE obtained from twenty biological replicates. ** are significantly different at *P* < 0.01 (LSD test).

To determine whether the overexpression of the *LcCHI2* gene could phenocopy the stress tolerances of transgenic tobacco in monocotyledons crops, we produced transgenic maize plants harboring the *pTF101-pZmUBI-LcCHI2-tNOS* construct. DNAs were extracted from leaves of T_0_ generation transgenic plants and use to detect the integration of target sequence into genomes of transgenic plants by PCR. The results showed that six transgenic events harbored both the selection marker and *LcCHI2* genes (Supplementary Figure S7). Southern blot with bar gene as the probe showed that transgenic lines Ox2, Ox4, Ox7, and Ox10 were single copy while the transgenic lines Ox5 and Ox12 contained three copies of target gene (Supplementary Figure S8). Reverse-transcriptional PCR (RT-PCR) and chitinase activity assay indicated that three representative transgenic lines accumulated abundance *LcCHI2* transcripts and enhanced chitinase activities compared to null plants with the absence of *LcCHI2* (Figures 5A,B). Then these transgenic plants were grown in normal and alkaline soils in the greenhouse (Supplementary Figure S9). There are no significant different between the growth of transgenic (Ox) and their respective non-transgenic plants in normal soil (null). However, the transgenic plants were healthier than the corresponding null plants in alkaline soil. A significant increased plant height and SPAD value were observed in transgenic plants compared their null plants (Supplementary Figures S9B,C). To further evaluate the effects of divergent salt stress on the transgenic maize, 2 week-old plants were watered with 200 mM NaCl. As expected, no significant differences were observed between the growth of transgenic (Ox) and their respective null plants. In contrast, the overexpression plants showed resistance to NaCl other than the null plants after 6 days treatment (Figures 6A,B). Although NaCl treatments decreased the plant height and SPAD value in both overexpression and null plants, the magnitudes of the overexpression plants were significantly lower than null plants (Figure 6B).

**FIGURE 7:**
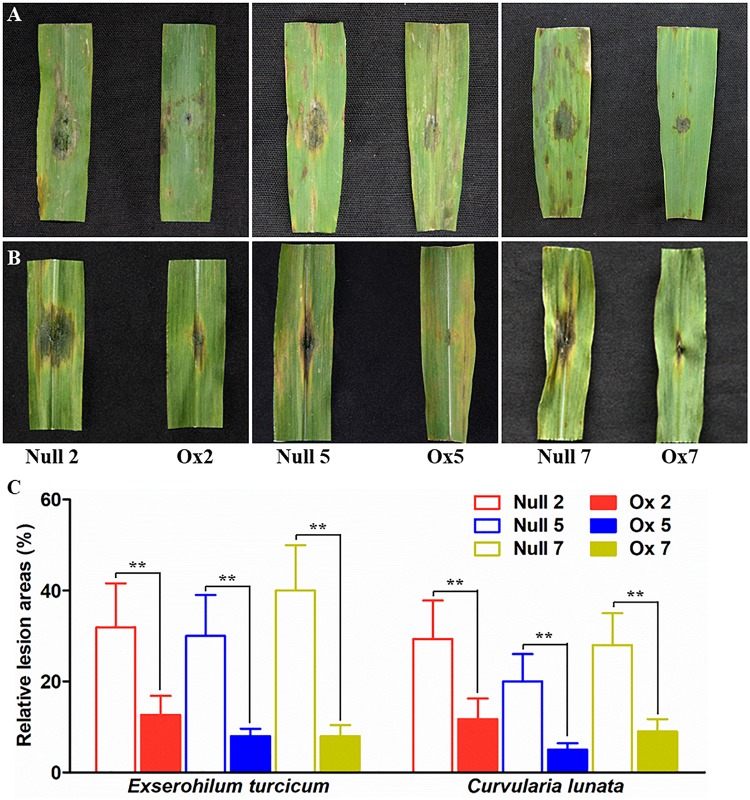
Pathogenesis analysis of null and *LcCHI2*-overexpressed maize. **(A)** Resistance of transgenic maize to fungal pathogen *Exserohilum turcicum*. **(B)** Responses of transgenic maize to the fungal pathogen *curvularia lunata* (Wakker) Boed. **(C)** The average lesion area of each independent transgenic line was calculated and their relative lesion areas are shown in columns after comparison with the average lesion area on wild-type tobacco. The transgenic events with HiII background were backcross to local commercial inbred line X923-1. The segregated positive and negative BC5F2 plants were used for the treatment. Values are the mean ± SE obtained from twenty biological replicates. ** are significantly different at *P* < 0.01 (LSD test).

Biotic resistances of the transgenic plants were also measured by infection the maize with two major fungal pathogens *Exserohilum turcicum* and *curvularia lunata* (Wakker) Boed, respectively. Typical necrosis symptoms were observed in the null plants while transgenic lines had significantly smaller lesion area after infection by *Exserohilum turcicum* or *curvularia lunata* (Wakker) Boed (Figures 7A–C). These results indicated that the overexpression of *LcCHI2* in maize significantly inhibited the growth of fungal pathogens.

To evaluate the effects of *LcCHI2* overexpression in agronomic traits, the gene in the transgenic events with HiII background (CHI-Ox2, CHI-Ox5, and CHI-Ox7) were backcross to local commercial inbred line X923-1. The genetically segregated positive and negative BC5F2 plants were crossed, respectively, with Y822 to produce hybrid seeds. Agronomic traits were measured from the hybrid lines in two replicated experiments. The results (Supplementary Table S1) showed that all transgenic events exhibited statistically no negative impact such as cob weight, cob diameter, ear kernel weight and ear diameter, indicating that overexpression of *LcCHI2* has no penalty on the growth and yield in the field conditions comparing with the controls.

## Discussion

In this study, the full-length cDNA of a type II chitinase gene was cloned and named as *LcCHI2*, which was first identified from an EST library in *Leymus Chinensis* under Na_2_CO_3_ stress (Jin et al., 2006). The cDNA-deduced protein sequence analysis indicated that the LcCHI2 contains two conserved domains including the signature 1 (PS00773) and signature 2 (PS00774) of glycosyl hydrolase 19 family (Supplementary Figure S2). Protein cluster analysis indicates that LcCHI2 belongs to class II chitinase (Supplementary Figure S1). Several members of the class II chitinase proteins, such as AtPR3 and pcht28, were reported to have the typical chitinase activity and overexpression of those chitinase genes could increase chitinase activity in transgenic plants (Verburg and Huynh, 1991; Chen et al., 1994; Tabaeizadeh et al., 1999). In this study, a significantly increased chitinase activity was positively correlated with the up-regulation of *LcCHI2* in *Leymus Chinensis* under both NaCl and Na_2_CO_3_ stress condition. Moreover, the transgenic tobacco and maize plants that overexpressed *LcCHI2* also showed higher chitinase activity than control plants. Interestingly, protein alignment indicated that LcCHI2 and its homologs contain an N terminal secretary signal peptide (Supplementary Figure S3B). These results supported that LcCHI2 is a secreted active chitinase in *Leymus Chinensis*.

Different types of chitinases were induced by differential pathogen attack and have been characterized as pathogenesis-related (PR) proteins (van Loon et al., 2006; Kesari et al., 2015). Phylogenic analysis showed that LcCHI2 is a homolog of several reported PR3 proteins, including TaPR3, HvPR3, CaCHI2, pcht28, and AtPR3 (Supplementary Figure S3) (Verburg and Huynh, 1991; Tabaeizadeh et al., 1999; Hong and Hwang, 2002; Shin et al., 2008; Scheler et al., 2016). These PR3 proteins were proposed to be involved in plant defense responses. Plant chitinases were thought to degrade the major structural polysaccharide of fungal cell walls in the intercellular space to limit fungal growth (Brogue et al., 1991). The purified AtPR3 protein showed antifungal chitinase activity and inhibited the growth of *Trichoderma reesei in vitro* (Verburg and Huynh, 1991). The overexpression of chitinase resulted in enhanced resistance in tomato, rice, Italian ryegrass and grapevine to *Verticillium dahlia*, Magnaporthe *grisea*, *Puccinia coronate*, and *Uncinula necator*, respectively (Nishizawa et al., 1999; Tabaeizadeh et al., 1999; Yamamoto et al., 2000; Takahashi et al., 2005). Moreover, expression of a barley *PR3* chitinase gene in transgenic wheat resulted in enhanced resistance to infection by *Erysiphe graminis*, *Blumeria graminis*, *Pucinia recondita*, and *Fusarium graminearum* (Oldach et al., 2001; Shin et al., 2008). To elucidate the defense response of *LcCHI2*, we investigated the biological functions of the *LcCHI2* in transgenic tobacco and maize. The overexpression of *LcCHI2* significantly enhanced resistance to multiple pathogens in tobacco and maize. The transgenic tobacco plants also showed resistance to a bacterial pathogen *Pseudomonas tabaci Stevens*, which do not have chitin or related structure in their cell wall. A similar phenomenon was observed in the transgenic *Arabidopsis* when overexpressing *CaCHI2* gene (Hong and Hwang, 2006). In fact, chitinase expression in plant tissues is strongly induced by infection with viruses and bacteria in addition to fungal pathogen (van Loon et al., 2006). Therefore, the PR3 type protein may inhibit the pathogen invasion by an unknown mechanism other than directly degrading the chitin oligomers.

In addition to biotic stress, a number of cluster II chitinases were reported to be involved in developmental and various abiotic responses. The ScCHT46 showed 92% protein identify with LcCHI2 and encode a chitinase-antifreeze protein in winter rye (Yeh et al., 2000). The expression of *ScCHT46* and its homologs in winter wheat, *HvPR3*, are responsive to cold and drought (Yeh et al., 2000). Moreover, it is reported that NaCl, drought and Mannitol induces the expression of several class II chitinases, including *CaCHI2*, *pcht28*, and *AtPR3* (Chen et al., 1994; Hong and Hwang, 2002, 2006; Seo et al., 2008). The overexpression of *CaCHI2* increased the osmotic stress in *Arabidopsis* (Hong and Hwang, 2006). In contrast, a mutation of *AtPR3* affected the seeds geminating under high salt (Seo et al., 2008). Similarly, our results showed that the expression of *LcCHI2* was up-regulated by Na^+^ stress conditions including treatment with NaCl and Na_2_CO_3_. Moreover, the activity of chitinase was increased under NaCl and Na_2_CO_3_ stresses in *Leymus chinensis*. Taking together, these results suggested that *LcCHI2* play an important role in abiotic stress tolerance similar to its homologs in other plants.

Interestingly, the overexpression of *LcCHI2* showed a significant tolerance to NaCl and Na_2_CO_3_ treatments in both tobacco and maize but not to the osmotic or drought stresses. These results implicated that the overexpression of *LcCHI2* might assist to reduce Na^+^ ionic toxicity other than osmotic stress or improving water-use-efficiency. Coincidence with this assumption, the Na^+^ contents were decreased in the transgenic tobacco under NaCl treatment. Recently, it is proposed that the amount of the carboxyl groups on de-methesterified pectin could bind cations such as Na^+^ and sequester more Na^+^ in the cell wall, which may contribute to salt stress resistance in plants (An et al., 2014; de Lima et al., 2014; Byrt et al., 2018). However, we did not detect any significant difference in the pectin content between transgenic and WT plants (data not show). Instead, increased cellulose and decreased hemicelluloses were observed in the overexpression lines compared with WT plants under salt treatment condition (Supplementary Figure S10). This phenomenon is reminiscent of the Na^+^ sensitive mutant *Atctl1*, which affects the contents of cellulose and extractability of hemicelluloses (Hermans et al., 2011; Sanchez-Rodriguez et al., 2012). It is reported that mutation of *AtCTL1* increased the Na^+^ influx rather than defects in Na^+^ efflux activity (Kwon et al., 2007). Therefore, the overexpression of the *LcCHI2*, a homolog of *AtCTL1* (Supplementary Figure S2), may have an opposite effect and inhibit the Na^+^ influx, which could explain the deceased Na^+^ content in *LcCHI2* transgenic plants.

Although cell wall metabolism is rationally correlated with salt stress, the exact mechanism is still largely unknown (Le Gall et al., 2015). Mutation of *AtCES8* (cellulose synthase 8) or *AtCSLD5* (cellulose synthase-like 5) resulted tolerance or hypersensitive to salt stress, respectively (Chen et al., 2005; Zhu et al., 2010). Moreover, mutation the chitinase-like gene *AtCTL1* reduced the cellulose content and led to salt-sensitive of *Arabidopsis* (Kwon et al., 2007; Sanchez-Rodriguez et al., 2012). Compared with dicot, there has been no report on the modification of cell wall compound contributing to salt tolerance in grass. In this study, the overexpression of *LiCHI2* in both tobacco and maize enhanced the salt and pathogen stress tolerances, suggesting similar roles of the chitinase in dicot and grass. Although the overall architectures of plant cell walls are similar in that they both consist of a network of cellulose fibers surrounded by a matrix of non-cellulosic polysaccharides, the types and abundance of non-cellulosic polysaccharides are significant different between grass and dicot cell walls (Vogel, 2008). It seems that cellulose represents the common component in plant cell walls and plays a pivotal role in regulating the salt stress. The changed cellulose content may regulate cross-linking and rigidity of the cell wall, which acts as a barrier for salt entrance or pathogen invasion.

Although a large number of alkali-responsive genes were reported from different plants, a few have been cloned and characterized (Zhao et al., 2016). Overexpression of the alkali-stress-inducable RMtATP6 and NADP-ME_2_ in rice and *Arabidopsis* enhanced the tolerance against osmotic, NaCl and Na_2_CO_3_ stresses (Zhang et al., 2006; Liu et al., 2007). Transgenic rice with *PtNHA1* and *PutNHX* genes, which were cloned from an alkaline soil plant, increased tolerance of shoots to NaCl and roots to NaHCO_3_, respectively (Kobayashi et al., 2012). It is noted that all these studies were conducted in nutrient solution or agar plate in laboratory. In contrast, we presented the performance of the transgenic maize with natural alkaline soil or treated soil (Supplementary Figure S9). Moreover, the transgenic maize showed no penalty on growth and yield in field condition (Supplementary Table S1). Therefore, we proposed that overexpression of *LcCHI2* represent a potential strategy for engineering biotic and alkaline resistance plants.

## Data Availability Statement

All datasets generated for this study are included in the article/Supplementary Material.

## Author Contributions

XL, CW, and DH planned and designed the research, and wrote the manuscript. XL, YY, QL, and SD performed the tobacco experiments. XL, XJ, YJY, JG, NL, and YL performed the maize experiments. SH was in charge of the green house management.

## Conflict of Interest

The authors declare that the research was conducted in the absence of any commercial or financial relationships that could be construed as a potential conflict of interest.

## Supplementary Material

FIGURE S1 Construct maps of the recombinant plasmids for transformation in tobacco **(A)** and maize **(B)**.

FIGURE S2 Phylogenetic tree constructed with class II chitinase sequences retrieved by BLAST in the NCBI database.

FIGURE S3 **(A)** Protein similarity of LcCHI2 with its homologs. **(B)** Alignment of deduced amino acid sequences of LcCHI2 with its homologs from other plant species.

FIGURE S4 Morphology of the *LcCHI2*-overexpressing transgenic tobacco plants under normal growth condition.

FIGURE S5 Drought **(A)**, neutral salt **(B)**, and alkaline salt **(C)** stress tolerance assays of wild-type and LcCHI2 overexpression tobacco plants grow in soil.

FIGURE S6 MDA contents and relative electrical conductivity of wild-type and *LcCHI2*-transgenic tobacco plants under 200 mM NaCl treatment. ** are significantly different at *P* < 0.01 (LSD test).

FIGURE S7 PCR detection of selection marker **(A)** and *LcCHI2* genes **(B)** in transgenic maize plants.

FIGURE S8 Southern blot of transgenic lines in maize.

FIGURE S9 Growth performance **(A)**, plant height **(B)**, and SPAD value **(C)** of transgenic maize in normal and alkaline soils. Columns with different small letters are significantly different at *P* < 0.05.

FIGURE S10 Cellulose and hemicelluloses contents in wild-type and *LcCHI2*-transgenic tobacco plants under 200 mM NaCl treatment.

TABLE S1 Agronomic traits of transgenic maize grow in the field condition.

## References

[B1] An P. Li X. Zheng Y. Matsuura A. Abe J. Eneji A. E. (2014). Effects of NaCl on root growth and cell wall composition of two soya bean cultivars with contrasting salt tolerance. *J. Agron. Crop Sci.* 200 212–218. 10.1111/jac.12060

[B2] Brogue K. Chet I. Holliday M. Cressman R. Biddle P. Knowlton S. (1991). Transgenic plants with enhanced resistance to the fungal pathogen *Rhizoctonia solani*. *Science* 254 1194–1197. 10.1126/science.254.5035.1194 17776411

[B3] Byrt C. S. Munns R. Burton R. A. Gilliham M. Wege S. (2018). Root cell wall solutions for crop plants in saline soils. *Plant Sci.* 269 47–55. 10.1016/j.plantsci.2017.12.012 29606216

[B4] Chen R. D. Yu L. X. Greer A. F. Cheriti H. Tabaeizadeh Z. (1994). Isolation of an osmotic stress-induced and abscisic-acid-induced gene encoding an acidic endochitinase from Lycopersicon-Chilense. *Mol. Gen. Genet.* 245 195–202. 10.1007/bf00283267 7816027

[B5] Chen Z. Hong X. Zhang H. Wang Y. Li X. Zhu J. K. (2005). Disruption of the cellulose synthase gene, AtCesA8/IRX1, enhances drought and osmotic stress tolerance in *Arabidopsis*. *Plant J.* 43 273–283. 10.1111/j.1365-313X.2005.02452.x 15998313

[B6] de Lima R. B. dos Santos T. B. Vieira L. G. de Lourdes Lucio Ferrarese M. Ferrarese-Filho O. Donatti L. (2014). Salt stress alters the cell wall polysaccharides and anatomy of coffee (*Coffea arabica* L.) leaf cells. *Carbohydr. Polym.* 112 686–694. 10.1016/j.carbpol.2014.06.042 25129798

[B7] Frame B. R. Shou H. Chikwamba R. K. Zhang Z. Xiang C. Fonger T. M. (2002). *Agrobacterium tumefaciens*-mediated transformation of maize embryos using a standard binary vector system. *Plant Physiol.* 129 13–22. 10.1104/pp.000653 12011333 PMC1540222

[B8] Hermans C. Porco S. Vandenbussche F. Gille S. De Pessemier J. Van Der Straeten D. (2011). Dissecting the role of CHITINASE-LIKE1 in nitrate-dependent changes in root architecture. *Plant Physiol.* 157 1313–1326. 10.1104/pp.111.181461 21949212 PMC3252165

[B9] Hoekema A. Hirsh P. R. Hooykaas P. J. Schilperoort R. A. (1983). A binary plant vector strategy based on separation of vir-and T-region of the *Agrobacterium tumefaciens* Ti-plasmid. *Nature* 303 179–180. 10.1038/303179a0

[B10] Hong J. K. Hwang B. K. (2002). Induction by pathogen, salt and drought of a basic class II chitinase mRNA and its in situ localization in pepper (*Capsicum annuum*). *Physiol. Plant.* 114 549–558. 10.1034/j.1399-3054.2002.1140407.x 11975728

[B11] Hong J. K. Hwang B. K. (2006). Promoter activation of pepper class II basic chitinase gene, CAChi2, and enhanced bacterial disease resistance and osmotic stress tolerance in the CAChi2-overexpressing *Arabidopsis*. *Planta* 223 433–448. 10.1007/s00425-005-0099-6 16151843

[B12] Jin H. Plaha P. Park J. Y. Hong C. P. Lee I. S. Yang Z. H. (2006). Comparative EST profiles of leaf and root of *Leymus chinensis*, a xerophilous grass adapted to high pH sodic soil. *Plant Sci.* 170 1081–1086. 10.1016/j.plantsci.2006.01.002

[B13] Kesari P. Patil D. N. Kumar P. Tomar S. Sharma A. K. (2015). Structural and functional evolution of chitinase-like proteins from plants. *Proteomics* 15 1693–1705. 10.1002/pmic.201400421 25728311

[B14] Kikuchi T. Masuda K. (2009). Class II chitinase accumulated in the bark tissue involves with the cold hardiness of shoot stems in highbush blueberry (*Vaccinium corymbosum* L.). *Sci. Hortic.* 120 230–236. 10.1016/j.scienta.2008.11.007

[B15] Kobayashi S. Abe N. Yoshida K. T. Liu S. Takano T. (2012). Molecular cloning and characterization of plasma membrane- and vacuolar-type Na(+)/H(+) antiporters of an alkaline-salt-tolerant monocot, *Puccinellia tenuiflora*. *J. Plant Res.* 125 587–594. 10.1007/s10265-012-0475-9 22270695

[B16] Kwon Y. Kim S. H. Jung M. S. Kim M. S. Oh J. E. Ju H. W. (2007). *Arabidopsis* hot2 encodes an endochitinase-like protein that is essential for tolerance to heat, salt and drought stresses. *Plant J.* 49 184–193. 10.1111/j.1365-313X.2006.02950.x 17156413

[B17] Le Gall H. Philippe F. Domon J. M. Gillet F. Pelloux J. Rayon C. (2015). Cell wall metabolism in response to abiotic stress. *Plants (Basel)* 4 112–166. 10.3390/plants4010112 27135320 PMC4844334

[B18] Liu S. K. Cheng Y. X. Zhang X. X. Guan Q. J. Nishiuchi S. Hase K. (2007). Expression of an NADP-malic enzyme gene in rice (*Oryza sativa*. L) is induced by environmental stresses; over-expression of the gene in *Arabidopsis* confers salt and osmotic stress tolerance. *Plant Mol. Biol.* 64 49–58. 10.1007/s11103-007-9133-3 17245561

[B19] Liu X. G. Yu Y. Liu Q. Deng S. Jin X. B. Yin Y. (2019). A Na_2_CO_3_-responsive chitinase gene from Chinese wildrye improve pathogen resistance and saline-alkali stress tolerance in transgenic tobacco and maize. *BioRxiv*[Preprint] 10.3389/fpls.2020.00504 PMC7198794 32411170

[B20] Nakamura T. Ishikawa M. Nakatani H. Oda A. (2008). Characterization of cold-responsive extracellular chitinase in bromegrass cell cultures and its relationship to antifreeze activity. *Plant Physiol.* 147 391–401. 10.1104/pp.106.081497 18359848 PMC2330313

[B21] Nishizawa Y. Nishio Z. Nakazono K. Soma M. Nakajima E. Ugaki M. (1999). Enhanced resistance to blast (*Magnaporthe grisea*) in transgenic Japonica rice by constitutive expression of rice chitinase. *Theor. Appl. Genet.* 99 383–390. 10.1007/s001220051248 22665169

[B22] O’Brien M. Colwell R. R. (1987). A rapid test chitinase activity that uses 4-methylumbelliferyl-N-acetyl-β-D-glucosaminide. *Appl. Environ. Microb.* 53 1718–1720. 10.1128/aem.53.7.1718-1720.1987 3662513 PMC203942

[B23] Oldach K. H. Becker D. Lorz H. (2001). Heterologous expression of genes mediating enhanced fungal resistance in transgenic wheat. *Mol. Plant Microbe Interact.* 14 832–838. 10.1094/MPMI.2001.14.7.832 11437256

[B24] Patil R. S. Ghormade V. Deshpande M. V. (2000). Chitinolytic enzymes: an exploration. *Enzyme Microb. Tech.* 26 473–483. 10.1016/S0141-0229(00)00134-4 10771049

[B25] Sanchez-Rodriguez C. Bauer S. Hematy K. Saxe F. Ibanez A. B. Vodermaier V. (2012). Chitinase-like1/pom-pom1 and its homolog CTL2 are glucan-interacting proteins important for cellulose biosynthesis in *Arabidopsis*. *Plant Cell* 24 589–607. 10.1105/tpc.111.094672 22327741 PMC3315235

[B26] Scheler B. Schnepf V. Galgenmuller C. Ranf S. Huckelhoven R. (2016). Barley disease susceptibility factor RACB acts in epidermal cell polarity and positioning of the nucleus. *J. Exp. Bot.* 67 3263–3275. 10.1093/jxb/erw141 27056842 PMC4892720

[B27] Seo P. J. Lee A. K. Xiang F. N. Park C. M. (2008). Molecular and functional profiling of *Arabidopsis* pathogenesis-related genes: insights into their roles in salt response of seed germination. *Plant Cell Physiol.* 49 334–344. 10.1093/pcp/pcn011 18203731

[B28] Shin S. Y. Mackintosh C. A. Lewis J. Heinen S. J. Radmer L. Dill-Macky R. (2008). Transgenic wheat expressing a barley class II chitinase gene has enhanced resistance against *Fusarium graminearum*. *J. Exp. Bot.* 59 2371–2378. 10.1093/jxb/ern103 18467324 PMC2423652

[B29] Tabaeizadeh Z. Agharbaoui Z. Harrak H. Poysa V. (1999). Transgenic tomato plants expressing a Lycopersicon chilense chitinase gene demonstrate improved resistance to *Verticillium dahliae* race 2. *Plant Cell Rep.* 19 197–202. 10.1007/s002990050733 30754748

[B30] Takahashi W. Fujimori M. Miura Y. Komatsu T. Nishizawa Y. Hibi T. (2005). Increased resistance to crown rust disease in transgenic Italian ryegrass (*Lolium multiflorum* Lam.) expressing the rice chitinase gene. *Plant Cell Rep.* 23 811–818. 10.1007/s00299-004-0900-1 15599752

[B31] van Hengel A. J. Tadesse Z. Immerzeel P. Schols H. van Kammen A. de Vries S. C. (2001). N-acetylglucosamine and glucosamine-containing arabinogalactan proteins control somatic embryogenesis. *Plant Physiol.* 125 1880–1890. 10.1104/pp.125.4.1880 11299367 PMC88843

[B32] van Loon L. C. Rep M. Pieterse C. M. (2006). Significance of inducible defense-related proteins in infected plants. *Annu. Rev. Phytopathol.* 44 135–162. 10.1146/annurev.phyto.44.070505.143425 16602946

[B33] Verburg J. G. Huynh Q. K. (1991). Purification and characterization of an antifungal chitinase from *Arabidopsis thaliana*. *Plant Physiol.* 95 450–455. 10.1104/pp.95.2.450 16668004 PMC1077551

[B34] Vogel J. (2008). Unique aspects of the grass cell wall. *Curr. Opin. Plant Biol.* 11 301–307. 10.1016/j.pbi.2008.03.002 18434239

[B35] Wang Z. Q. Zhu S. Q. Yu R. P. Li L. Shan G. Z. (1993). *The Salinealkali Soil of China (In Chinese).* Beijin: The Science Press.

[B36] Yamamoto T. Iketani H. Ieki H. Nishizawa Y. Notsuka K. Hibi T. (2000). Transgenic grapevine plants expressing a rice chitinase with enhanced resistance to fungal pathogens. *Plant Cell Rep.* 19 639–646. 10.1007/s002999900174 30754799

[B37] Yeh S. Moffatt B. A. Griffith M. Xiong F. Yang D. S. Wiseman S. B. (2000). Chitinase genes responsive to cold encode antifreeze proteins in winter cereals. *Plant Physiol.* 124 1251–1264. 10.1104/pp.124.3.1251 11080301 PMC59223

[B38] Zhang X. X. Takano T. Liu S. K. (2006). Identification of a mitochondrial ATP synthase small subunit gene (RMtATP6) expressed in response to salts and osmotic stresses in rice (*Oryza sativa* L.). *J. Exp. Bot.* 57 193–200. 10.1093/jxb/erj025 16317034

[B39] Zhao Q. Suo J. W. Chen S. X. Jin Y. D. Ma X. L. Yin Z. P. (2016). Na2CO3-responsive mechanisms in halophyte *Puccinellia tenuiflora* roots revealed by physiological and proteomic analyses. *Sci. Rep.* 6:32717. 10.1038/Srep32717 27596441 PMC5011731

[B40] Zhong R. Kays S. J. Schroeder B. P. Ye Z. H. (2002). Mutation of a chitinase-like gene causes ectopic deposition of lignin, aberrant cell shapes, and overproduction of ethylene. *Plant Cell* 14 165–179. 10.1105/tpc.010278 11826306 PMC150558

[B41] Zhu J. H. Lee B. H. Dellinger M. Cui X. P. Zhang C. Q. Wu S. (2010). A cellulose synthase-like protein is required for osmotic stress tolerance in *Arabidopsis*. *Plant J.* 63 128–140. 10.1111/j.1365-313X.2010.04227.x 20409003 PMC3061338

