# Supplementary material for: A Na2CO3-Responsive Chitinase Gene From Leymus chinensis Improve Pathogen Resistance and Saline-Alkali Stress Tolerance in Transgenic Tobacco and Maize
Source: Front Plant Sci. 2020 Apr 28;11:504. doi: 10.3389/fpls.2020.00504 (PMC7198794; doi:10.3389/fpls.2020.00504)
Supplement: FIGURE S1 — Construct maps of the recombinant plasmids for transformation in tobacco (A) and maize (B). [file Data_Sheet_1.pdf]

Figure S1

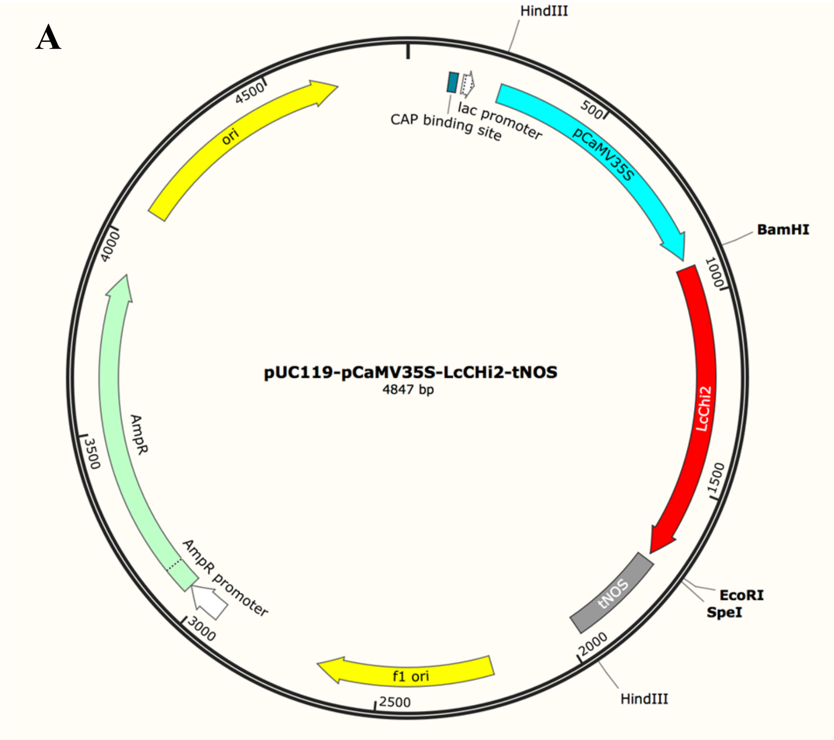
A
HindIII
CAP binding site
lac promoter
500
pCaMV35S
BamHI
1000
LcChi2
1500
EcoRI
SpeI
tNOS
2000
HindIII
2500
f1 ori
3000
AmpR promoter
3500
AmpR
4000
ori
4500
pUC119-pCaMV35S-LcCHi2-tNOS
4847 bp

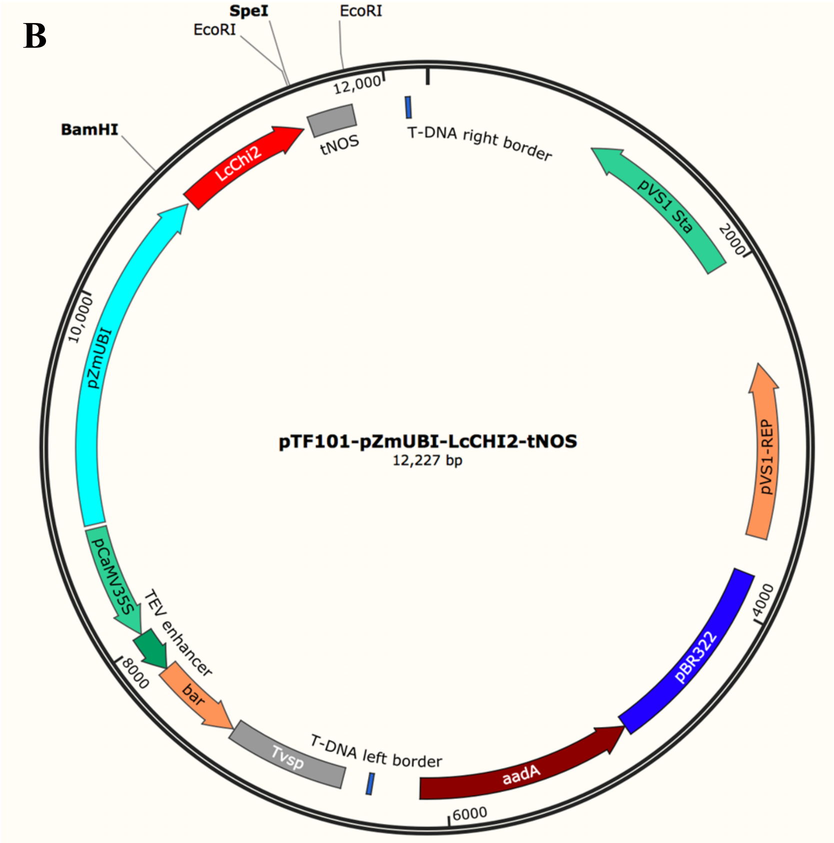
B
SpeI
EcoRI
EcoRI
12,000
T-DNA right border
tNOS
LcChi2
BamHI
pVS1 Sta
2000
10,000
pZmUBI
pTF101-pZmUBI-LcCHI2-tNOS
12,227 bp
pVS1-REP
pCaMV35S
TEV enhancer
4000
8000
bar
pBR322
Tvsp
T-DNA left border
aadA
6000

Figure S2

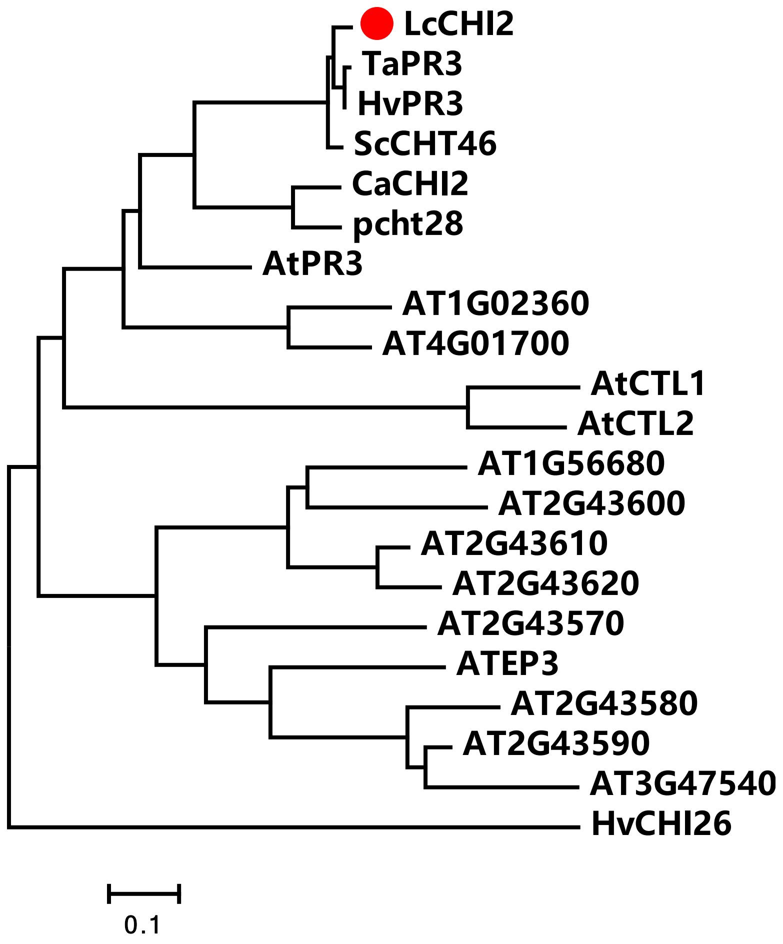
LcCHI2
TaPR3
HvPR3
ScCHT46
CaCHI2
pcht28
AtPR3
AT1G02360
AT4G01700
AtCTL1
AtCTL2
AT1G56680
AT2G43600
AT2G43610
AT2G43620
AT2G43570
ATEP3
AT2G43580
AT2G43590
AT3G47540
HvCHI26
0.1

Figure S3

A

Homolog \ Identity	LcCHI2	TaPR3	HvPR3	ScCHT46	CaCHI2
LcCHI2		95	95	92	57
TaPR3	98		98	93	57
HvPR3	97	98		94	57
ScCHT46	95	96	96		57
CaCHI2	71	71	71	71	

B

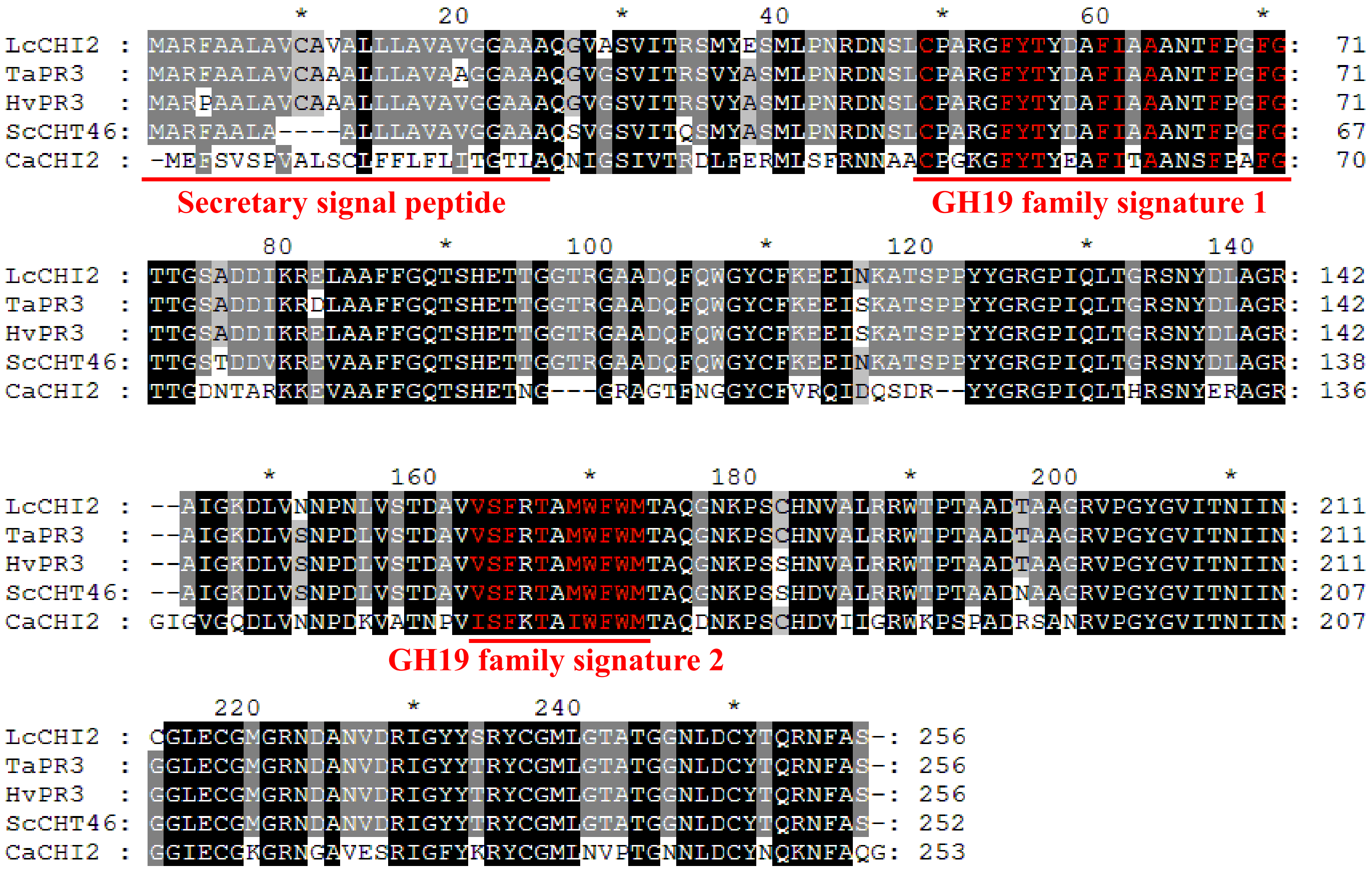

Figure S4

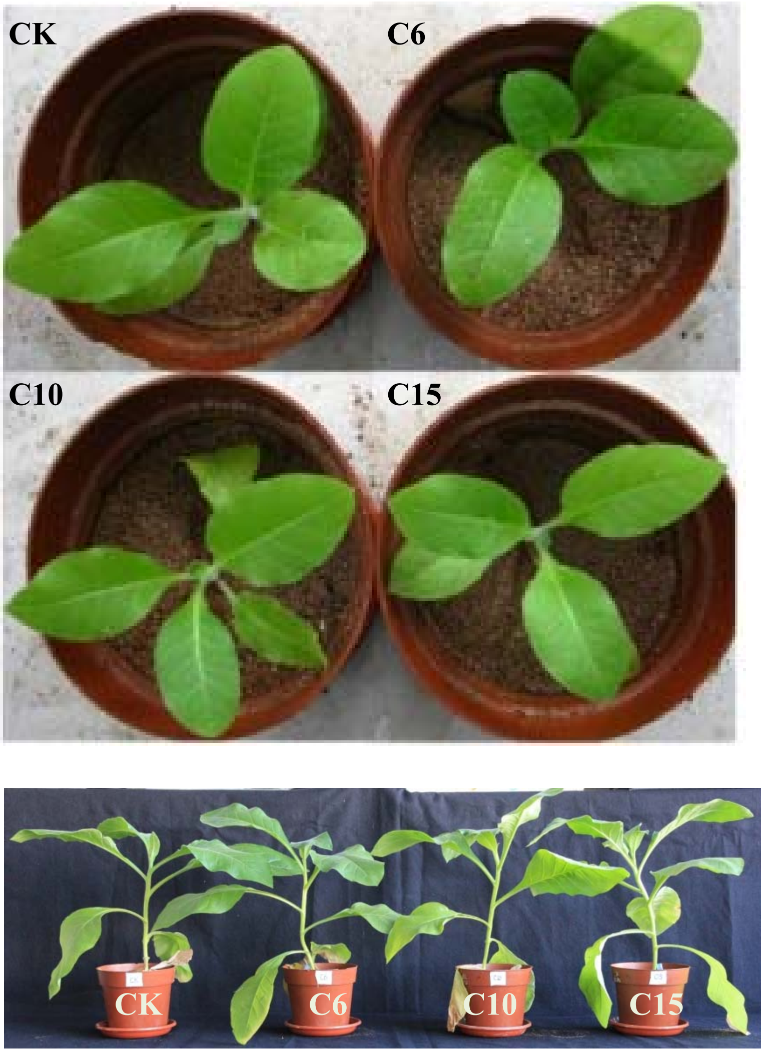

Figure S5

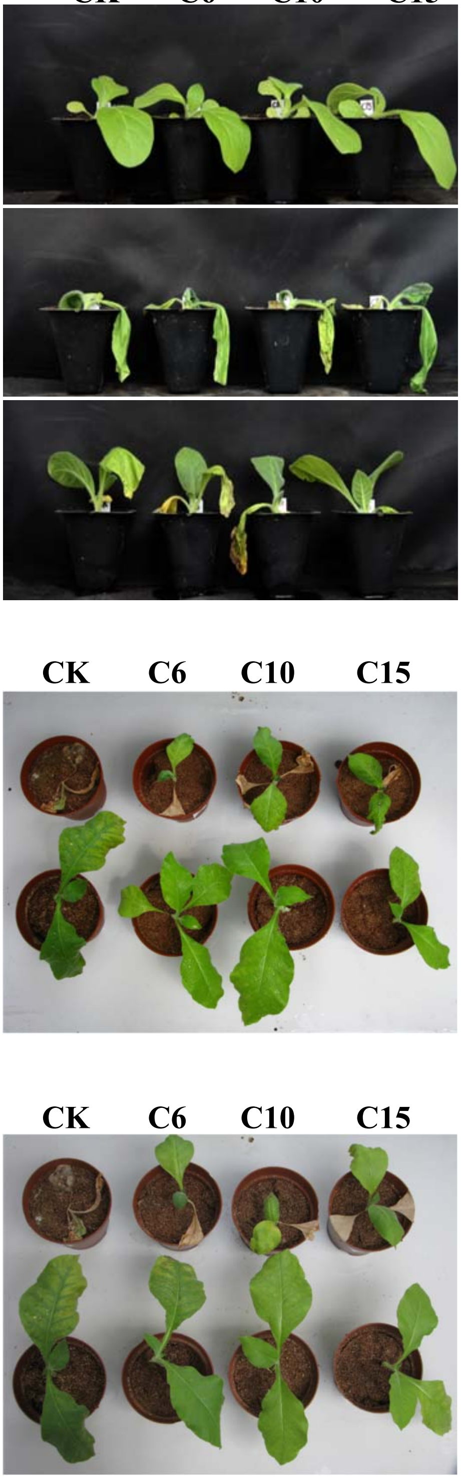
A
CK C6 C10 C15
Control
Drought (6d)
Re-water
B
CK C6 C10 C15
400 NaCl
Control
C
CK C6 C10 C15
100 mM Na_2C0_3
Control

Figure S6

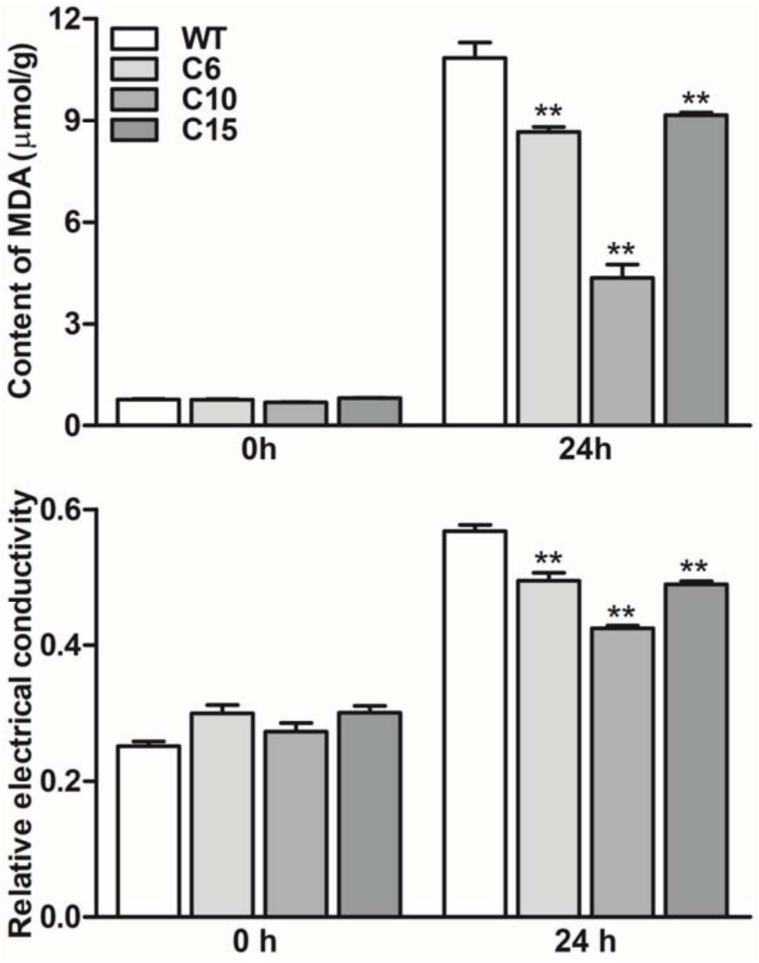
WT
C6
C10
C15
Content of MDA (μmol/g)
12
9
6
3
0
**
**
**
0h
24h
Relative electrical conductivity
0.6
0.4
0.2
0.0
**
**
**
0 h
24 h

Figure S7

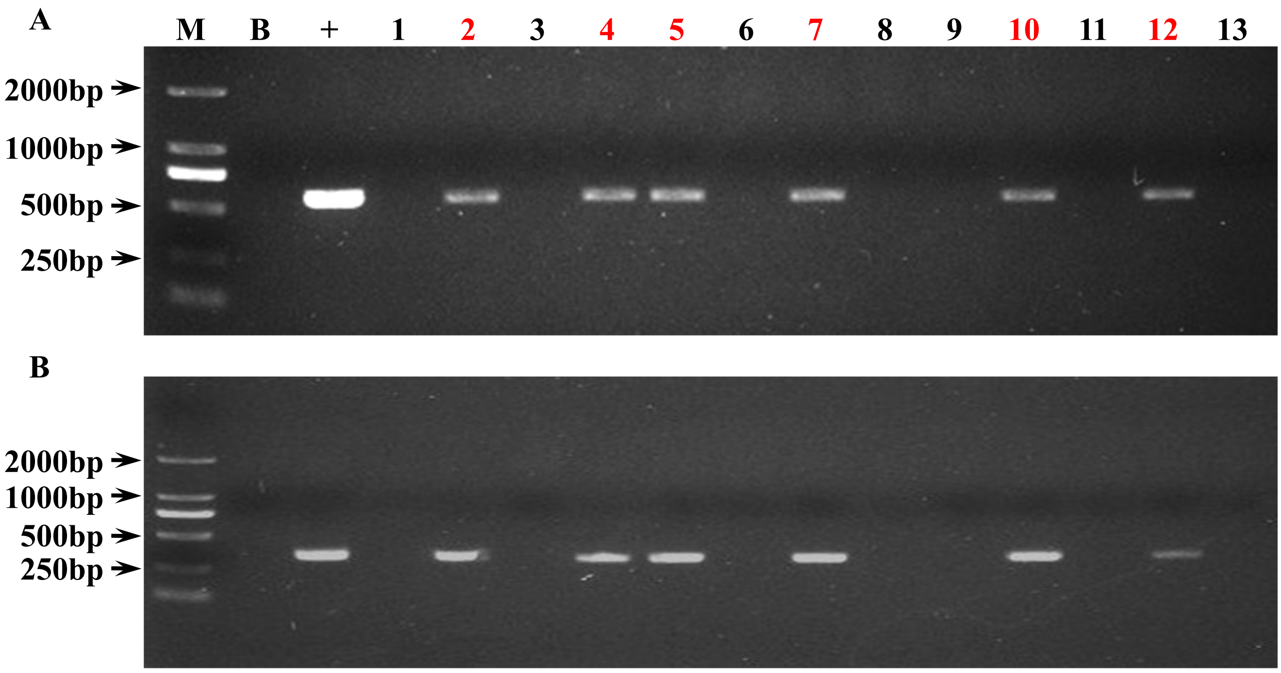
A
M
B
+
1
2
3
4
5
6
7
8
9
10
11
12
13
2000bp
1000bp
500bp
250bp
B
2000bp
1000bp
500bp
250bp

Figure S8

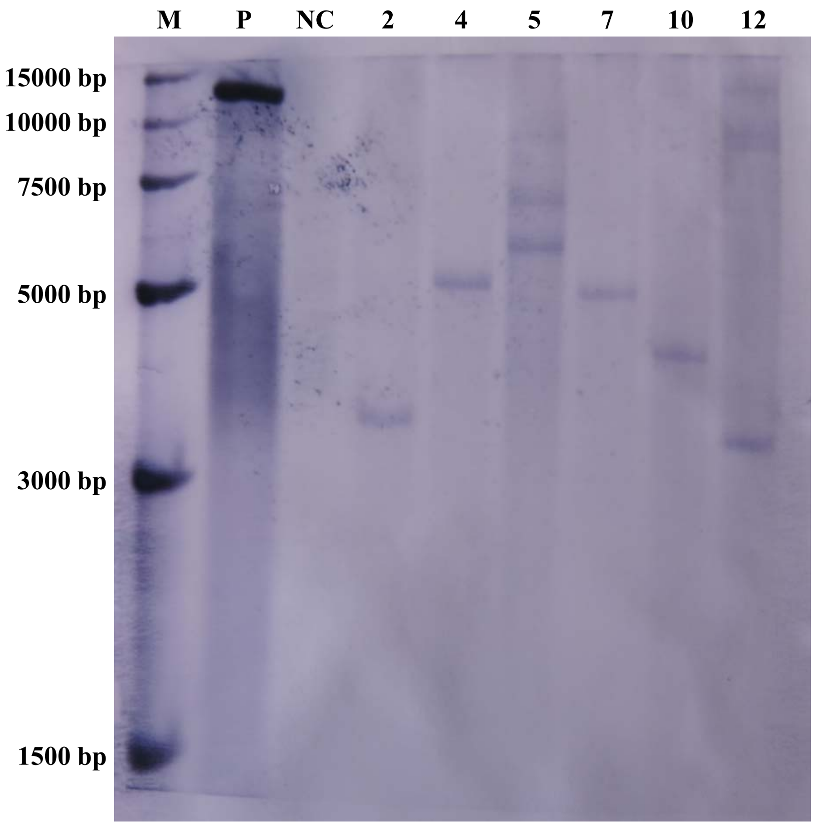
M
P
NC
2
4
5
7
10
12
15000 bp
10000 bp
7500 bp
5000 bp
3000 bp
1500 bp

Figure S9

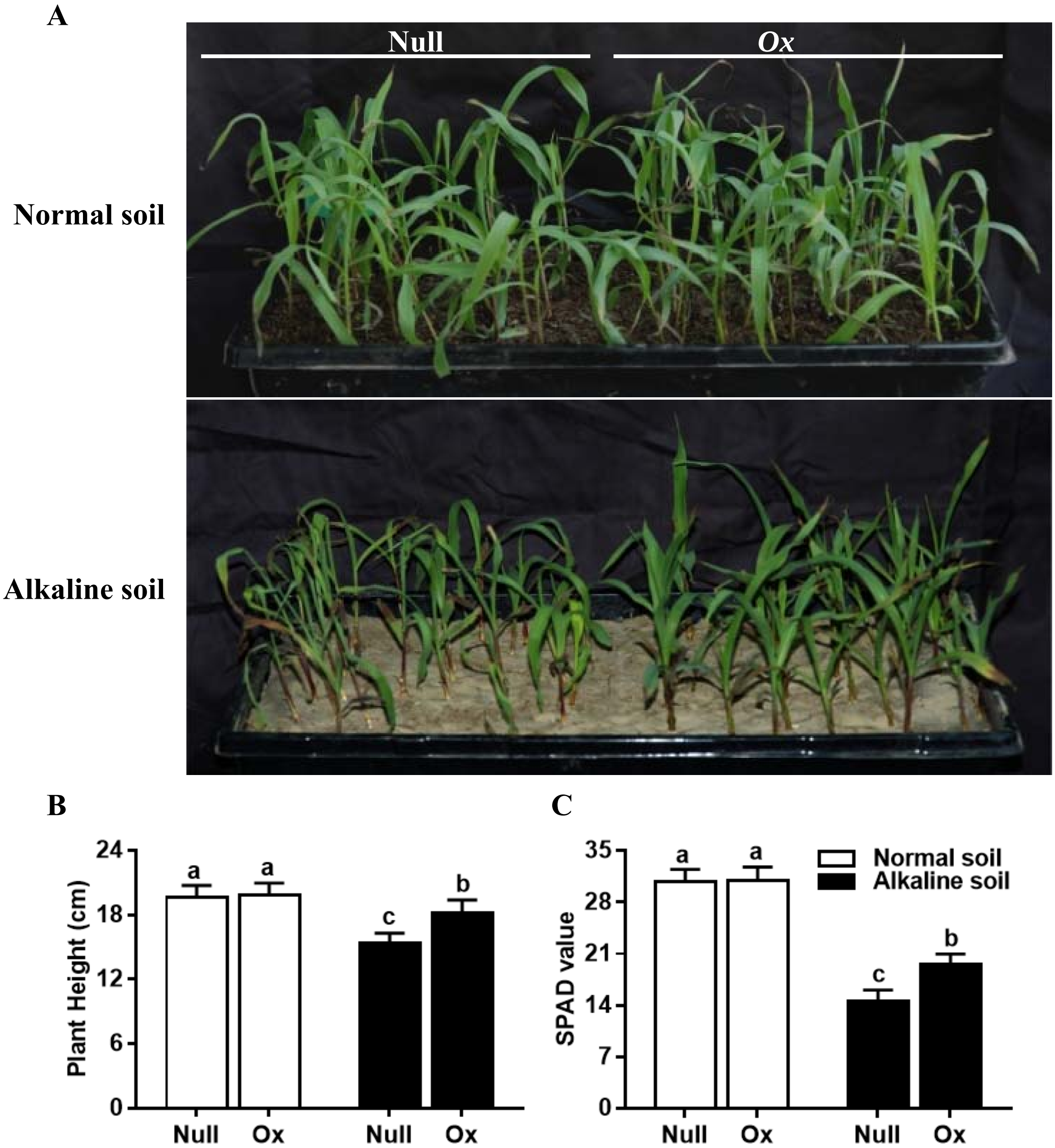
A
Null
Ox
Normal soil
Alkaline soil
B
Plant Height (cm)
24
18
12
6
0
a
a
c
b
Null
Ox
Null
Ox
C
SPAD value
35
28
21
14
7
0
a
a
c
b
Normal soil
Alkaline soil
Null
Ox
Null
Ox

Figure S10

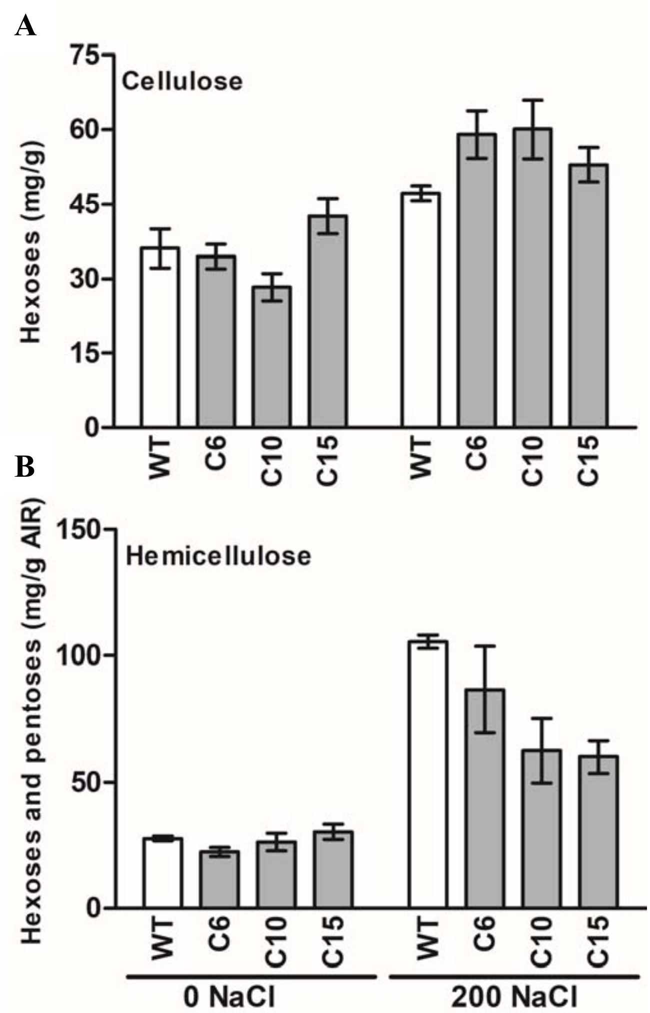
A
75
60
45
30
15
0
Cellulose
Hexoses (mg/g)
WT
C6
C10
C15
WT
C6
C10
C15
B
150
100
50
0
Hemicellulose
Hexoses and pentoses (mg/g AIR)
WT
C6
C10
C15
WT
C6
C10
C15
0 NaCl
200 NaCl

Table S1. Agronomic traits of transgenic maize grow in the field condition.

**Experiments**	Trait	Mean ± SD(Ox2)	Mean ± SD(Null2)	Mean ± SD(Ox5)	Mean ± SD(Null5)	Mean ± SD(Ox7)	Mean ± SD(Null7)
Field 1	Plant height (cm)	277.37±21.74	284.45±17.46	278.90±16.94	277.87±10.65	264.00±14.4	267.00±19.55
	Ear height (cm)	109.68±12.19	106.20±12.15	105.40±10.15	105.93±7.47	100.70±7.44	99.11±11.09
	Internodes above ear	6.52±0.61	6.55±0.60	6.70±0.57	6.73±0.59	6.85±0.37	6.67±0.59
	Internodes under ear	6.42±0.61	6.50±0.61	6.95±0.69	6.93±0.70	6.85±0.49	6.78±0.55
	Tassel branch number	2.32±0.89	1.95±1.00	2.60±1.43	2.80±1.15	3.30±1.53	3.28±1.45
	Cob diameter(mm)	26.04±0.99	26.30±0.88	26.22±1.20	25.11±1.58*	25.11±1.09	25.02±0.99
	Cob weight(g)	241.85±22.52	251.88±24.37	249.22±34.32	224.93±21.83*	219.04±31.98	206.26±40.42
	Ear length(cm)	19.98±0.99	20.71±1.19	19.98±1.86	19.91±1.22	19.19±1.37	19.29±1.68
	Ear diameter(mm)	48.65±1.46	48.58±1.95	49.70±1.44	47.77±1.37*	47.00±1.67	46.75±2.37
	Ear row number	16.00±0.97	16.00±1.68	16.56±1.65	16.40±1.72	15.10±1.21	16.00±1.00
	Ear Kernel weight(g)	212.89±19.98	222.93±22.12	220.75±32.13	200.29±20.51*	194.02±28.84	181.42±36.49
Field 2	Plant height (cm)	278.50±23.51	272.40±44.22	255.90±15.63	256.45±33.95	269.15±21.49	280.90±16
	Ear height (cm)	110.05±8.81	108.70±8.52	101.95±13.08	102.35±12.37	98.15±7.71	103.90±10.61
	Internodes above ear	6.35±0.75	5.80±1.77	6.50±0.51	6.20±1.32	6.70±0.47	6.60±0.52
	Internodes under ear	6.80±0.41	6.95±0.22	6.75±0.91	6.70±0.73	6.75±0.55	6.90±0.32
	Tassel branch number	1.70±0.86	2.05±0.89	2.60±1.47	2.40±1.90	3.30±1.45	3.70±1.49
	Cob diameter(mm)	25.23±1.06	25.17±0.81	25.30±1.21	25.85±1.06	25.41±1.08	26.32±0.94
	Cob weight(g)	225.07±34.11	234.42±33.40	235.18±41.22	242.99±37.17	235.13±21.47	222.45±21.27
	Ear length(cm)	19.88±1.45	20.76±1.53	19.94±1.81	19.61±1.73	19.62±1.14	18.72±1.08*
	Ear diameter(mm)	47.97±1.54	47.43±2.19	48.45±1.79	48.21±2.53	47.74±1.46	47.57±1.21
	Ear row number	16.00±1.53	15.22±1.40	15.68±1.53	16.63±1.20	15.58±1.42	16.20±0.43
	Ear Kernel weight(g)	198.16±30.28	206.41±29.53	207.34±38.77	203.20±37.49	209.62±15.52	196.39±18.67
